# Design and Development of a Low-Cost Wearable Glove to Track Forces Exerted by Workers in Car Assembly Lines

**DOI:** 10.3390/s19020296

**Published:** 2019-01-13

**Authors:** Leire Francés, Paz Morer, Maria Isabel Rodriguez, Aitor Cazón

**Affiliations:** Department of Mechanical Engineering—Industrial Design Area, University of Navarra, 20018 San Sebastian, Spain; pmorer@tecnun.es (P.M.); mirodriguez@tecnun.es (M.I.R.); acazon@tecnun.es (A.C.)

**Keywords:** smart glove, wearable technology, components integration, pressure sensors, conductive textiles

## Abstract

Wearables are gaining widespread use and technologies are making it possible to monitor human physical activity and behaviour as part of connected infrastructures. Many companies see wearables as an opportunity to enhance worker safety since they can monitor their workers’ activity in real life scenarios. One of the goals of this technology is to integrate existing electronic components, such as sensors or conductors, in order to create fully wearable systems. This integration is constrained not only by technical factors but also by user requirements and internal company standards. This paper considers such constraints and presents preliminary research for the design of a wearable glove as a new tool to track forces exerted by workers in car assembly lines. The objective of the glove is to measure forces and compare these to maximum forces already identified by the company. Thus, the main objectives are to: (1) integrate the components based on the requirements of the users and the context of application, and (2) provide a new tool that can be used “in situ” to track workers. This study was carried out in close collaboration with Volkswagen through a human-centred iterative design process. Thus, this paper presents the development of a wearable device glove based on a specific design methodology where both the human and technological aspects are considered.

## 1. Introduction

Wearable electronics first boomed in the 1960s [1], with major developments having taken place in the last ten years. Wearable technologies make it possible to monitor human physical activity and behaviour. In contrast with other technological devices, such as laptops and smartphones, wearables are worn on people’s bodies and are composed of sensors and other technological components that allow activities to be monitored. Thus, one of the main functions of a wearable in industrial frameworks is to track activity and capture useful data that can be analysed afterwards [2]. This has attracted many companies to wearable devices and has encouraged the creation of new start-ups.

Wearables have great potential for industrial application since they can be used to objectify information. Some wearables with an ergonomic focus have already been used to evaluate workers’ workplace tasks and improve their efficiency by enhancing safety.

Numerous studies have been carried out in relation to muscular activity. There are systems that track trunk muscular activity [3], others that track the activity of the neck-arm-chest group of muscles [4], and others that are more focused on the hand-wrist group [5,6]. From a review of these studies on wearable systems and their multiple applications, some have focused on hand-wearable systems [5,7], others are based on health monitoring and prognosis [8], and others have focused on measuring behavioural aspects [9,10]. There is also another group of studies based on the main components of wearables [11,12].

Regarding the studies found in the literature, there are very few that have specifically examined the industrial application of these devices [13,14]. Nevertheless, examples can be found in the marketplace, such as ProGlove [15], a smart glove to help automotive workers in logistics. There are also other products in various fields that can be used for training workers, such as Xsens MVN [16] or those developed by BAE systems [17]. Devices have also been designed to protect workers’ health, including the Smart Cap, which collects fatigue related data to increase worker safety in fields like mining or the transport industry, and the Vandrico [18] hat, which provides cooling for the comfort of athletes, firefighters, surgeons and pilots. Companies that provide wearable technology include TekSan [19], which has developed a system for tracking grip pressure and has conducted research focused on textile sensors based on conductive polymers [20].

Finally, wearables have also been used to increased companies’ productivity by introducing these devices in the value chain of other companies, such as the ones developed by cyber glove systems [21].

### 1.1. Musculoskeletal Disorders

A number of studies found in the literature clearly show the importance of tracking movements and forces to prevent work-related musculoskeletal disorders (WMSD). WMSD are injuries and disorders that affect the movement of the human body and the musculoskeletal system (i.e., muscles, tendons, ligaments, nerves, discs, blood vessels, etc.). One study on the hands of workers in car assembly lines reported the prevalence of WMSD in a truck assembly line, where 47% of WMSDs affected the hand-wrist group [22,23]. Additionally, other studies on musculoskeletal symptoms in car assemblies have reported that 46% of workers suffered from WMSDs in their hands-wrists [24].

### 1.2. Project Opportunity

In car manufacturing lines, workers are subjected to repetitive movements that can later result in WMSD. This affects the workers’ safety as well as company efficiency. The Volkswagen Group (VW) is one of the world’s leading manufacturers of automobiles and commercial vehicles and the largest carmaker in Europe. The company is on board with digitalization and the new trends in Industry 4.0, such as self-driving systems, e-mobility, connected vehicle concepts and digital transformation as can be seen on the Volkswagen web page. This transformation could also be applied to their car manufacturing assembly lines, where the company seeks to improve flexibility in production and ensure workers’ safety.

In car assembly lines, there is a significant number of the tasks that consist of placing pieces on the body of the vehicle; therefore, the hands are largely involved in these processes. The Volkswagen factory located in the region of Navarra (Spain) manufactures several models from beginning to end, from chassis mounting to rear gate placement in the last assembly line. Thus, there are multiple tasks done by hand where workers are subjected to repetitive movements. Volkswagen asked our team to focus on the plug placement in the chassis of the car and the rear gate mounting. Both tasks consist of a series of movements performed with the fingers and the palm of the hand.

Maximum force values for such tasks were provided by Volkswagen as input for the project. These values were obtained from the EAWS (Ergonomic Assessment Worksheet), which is a first level ergonomic analysis methodology developed by Darmstadt University, the University of Turin and MTM, based on the standards UNE 1005, and ISO 11226 and 11228 [25,26,27].

This study is therefore based on hand-related tasks and will focus on the design of a low-cost glove that integrates existing electronic components to create a fully wearable system for measuring the force exerted by workers as they attach different pieces during assembly tasks.

## 2. Materials and Methods

**Figure 1:**
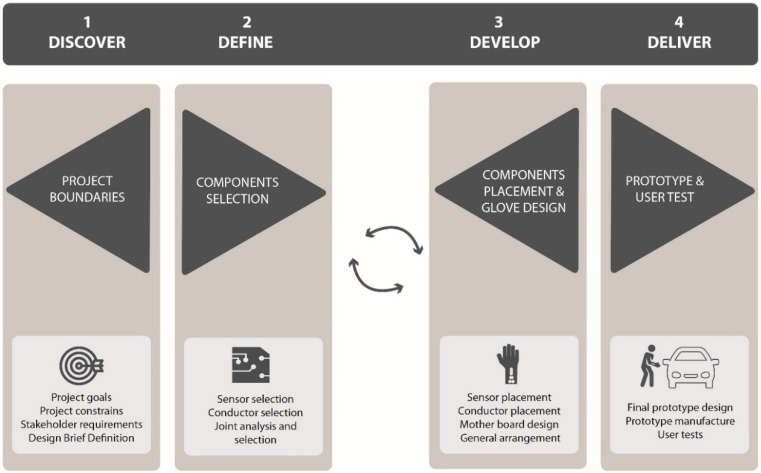
Smart glove design process based on the double diagram model (Adapted from Design Council).

The methodology used for this work was theoretical-practical and included human-centred design principles in all steps of the process (Figure 1). The design process is based on the Double Diamond Model [28], which is an iterative process. This means that ideas were developed, tested and redefined a number of times. In order to consider human factors, the iterative process was supported by the collaboration of stakeholders, and low-fidelity and final prototypes.

The Double Diamond Model consists of four distinct phases: discover, define, develop and deliver. The model was adapted to the design and development of a smart glove as follows:

- Discover Phase: This refers to the field work. In this phase, a qualitative exploration was first developed. Workers’ movements and characteristics of the target workstation were identified by observation. In order to fulfil this task properly, three design researchers from our team used written diaries to collect the observed data. Meetings were then organised with stakeholders. Based on findings from the exploration and conclusions from the meetings, the project goals and constraints, stakeholder constraints and the design brief were defined. Thus, the goal of this first phase was to determine the design brief.
- Define Phase: In this phase, the components were selected. First, sensors were selected and characterised. According to the sensor characteristics, different conductive materials were analysed and selected. After this, several sensor-conductive material joints were studied and the most suitable option was chosen. Thus, the goal of this first phase was to realise the component selection.
- Develop Phase: In this phase, component placement and the final glove arrangement were defined. After discovering and defining technological opportunities in previous phases, in the develop phase different components were arranged in the glove. In order to make design decisions, low-fidelity prototypes were constructed and different user tests were undertaken. First, the sensors were placed according to the experimental results. Based on the sensor placement, different component arrangements were analysed. After choosing the most suitable component arrangement, different circuitry options were analysed. Finally, on the basis of the selected circuitry, the motherboard was designed. Thus, the goal of this phase was to realise the component placement and a first glove design
- Delivery Phase: In this phase, prototypes were made and tested with different users. The aim of these prototypes was to test the functionality and the ergonomics in the lab and in a real scenario. In conclusion, the aim of the delivery phase was to have a prototype that can serve as a reference model for final products in the future. Thus, the goal of this last phase was to realise the prototype and testing.

As Figure 1 shows, the process is divided into four phases, and the interactions with users and prototypes are considered in each stage.

### 2.1. Discover Phase: Project Boundaries

This project aimed to design a prototype of a low-cost glove able to measure the forces applied by the operator’s hands, not only in laboratory environments, but also in real assembly lines.

**Figure 2:**
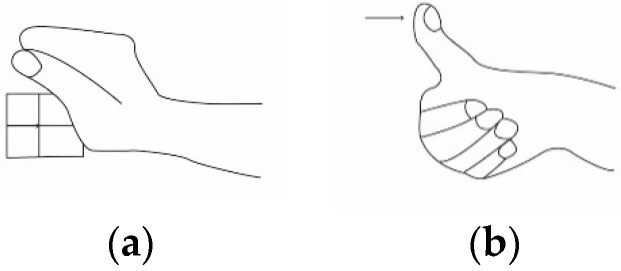
Hand-movements considered for this study. (**a**) Contact-grip (**b**) Distal compression.

Before defining the objectives and boundary conditions, the design team met the Volkswagen team, and a visit to the assembly lines was planned. During the visit, observations were made with written diaries. Three researchers participated at the same time in this observation stage. The diaries were designed previously and consisted of identifying worker behaviour and taking notes. As a result of the visit, the main movements involved in car assembly lines were identified and are shown in Figure 2.

As a result of the meetings and the visit, the main objectives were defined. In collaboration with Volkswagen it was decided to:

- Measure forces and record data for subsequent analysis on a PC.
- Embed the components in a model of a glove currently used on assembly lines for the protection of workers’ hands.
- Use low-cost components and manufacturing processes.
- Store the data on an SD card.
- Apply a User Centred Design strategy for the design of this glove.
- Measure two movements (efforts): contact grip and distal compression (Figure 2).

### 2.2. Define Phase: Component Selection

The main components were selected in the Define Phase. As mentioned in the Discovery Phase, Section 2.1, one of the main constraints of the project was having to embed the components into a glove that workers were actually using in car assembly lines. Thus, the component selection was based on the characteristics of the existing glove. It is a cotton glove with a polymer coating in the palm, which gives rigidity. The glove is flexible and adapts perfectly to the shape of the hand. First, the sensors were selected. Sensors must be linked to the main motherboard through the circuitry, so different conductive materials were then analysed and selected to create the circuitry. Finally, different joints between sensors and conductive materials, which are described in detail later in this paper (Section 2.2.2), were studied and the most suitable option was selected.

#### 2.2.1. Sensor Selection and Calibration

**Figure 3:**
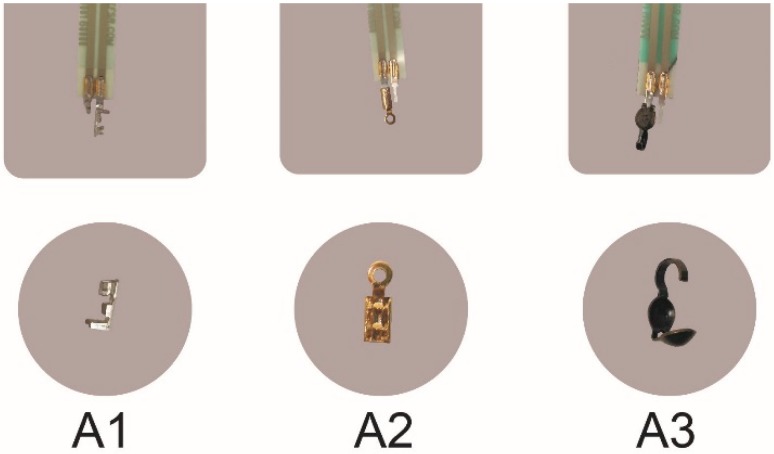
Different possibilities for crimped connections composed of different joints and sensors.

Since it was decided to track quantitative data using low-cost technology, Force-Sensitive Resistor (FSR)-Interlink model 402 sensors were selected. FSR sensors make it possible to detect physical loads between 0 N and 100 N. They are made of plastic and the connection tab is crimped on soft material. As for glove integration, the sensors are 12.5 mm in diameter, 56.77 mm long, 18.48 mm wide and 0.55 mm thick, and therefore appropriate to place on the fingertips or the palm of the hand. Moreover, they weigh 0.26 g, can easily be incorporated into the glove, and do not restrict hand movement. Different joint options are shown in Figure 3.

#### 2.2.2. Conductive Materials Selection

In order to select the conductive materials to connect the sensors with the controller, four different options were analysed:

- Woven Conductive Fabric: This woven conductive fabric is made of Copper+Nickel-plated nylon (Adafruit 1168). Small pieces are used for soft switches, plush keypads, capacitive touch sensors, and other textile interfaces. This highly conductive fabric has a resistance of less than 1 ohm per foot in any direction across the textile. Looking at the glove integration and flexibility, conductive fabric is normally used for tracking or making pressure areas to generate electricity. Since it is a fabric, it is very flexible and easy to integrate by stitching it into the glove. The disadvantage of the fabric is that once it is cut, it unravels, which means that small pieces of fabric are separated, and other glove parts could be short-circuited.
- Conductive Ribbons: Conductive ribbons are strong, and are composed of 316 L stainless steel with a 2.6 ohm/inch resistivity (Adafruit 1244). Depending on glove integration and flexibility, the ribbon is easy to integrate and stitch to both the glove and the woven conductive fabric. This is a great advantage for making common conductive tracks. Moreover, although flexible, the composition of the ribbon is robust and not easy to tear.
- Conductive Thread: The selected conductive thread was “stainless thin”, and is composed of 316 L stainless steel (Adafruit 640). It consists of two thin layers that can be stitched by hand or sewing machine. Its resistivity is 16 ohm/inch. The conductive thread is highly flexible and adaptable to the sewing machine, so strong joints can be made. The main advantage is the ability to make thin tracks in the part of the glove where conductive tracks have to be made independently.

Following these investigations, conductive thread was used to join the sensors with the rest of components. The conductive threads were also joined through conductive fabrics in common areas in order to simplify the circuitry. Finally, the joint of those common areas with the board was made with conductive ribbons. This configuration makes the circuit simpler and reduces the probability of short circuits.

#### 2.2.3. Sensor-Conductor Joint System Selection

After selecting the type of sensor and the conductive elements to transmit the signal from the sensors to the controller, the next step was how to join the two elements. This section details three different options: the crimped joint, crimped joint with soldering, and joining the sensor and conductor directly. Soldering options were dismissed due to incompatibility with the conductive thread. The resistance value is too high because the conductive thread has an element that differs from tin, and therefore they repel each other. In the same way, direct connection of the sensors and conductors (with no joint) were also dismissed due to the rigidity of the union. Thus, different crimped options were tested in order to select the most suitable joint. The options are shown in Figure 3.

The three options consist of different geometries that can be crimped in order to grip the sensors. The first option (A1) is made with a crimped joint and the sensor. The selected crimped joint is a horizontal rectangle and has a contact area of 6mm^2^ when it is crimped to the sensor. The second possibility (A2) consisted of a similar geometry but with a contact area of 8 mm^2^. The third option (A3) has a different geometry and is composed of two concave semi-spheres that can be pressed together. The contact area is 12 mm^2^ but due to the slight concavity, the contact area is not entirely accurate, and it is easy to remove the sensor. Thus, the joint selected for crimping the sensor was the second one (A2).

### 2.3. Develop Phase: Component Placement and Glove Arrangement

After selecting the main components, they were placed in the glove. The limitation factor is the sensors since their placement influences the general arrangement. To decide on the sensor placement, the literature was reviewed and an internal experiment was carried out. Once the sensors were placed, different configurations for the battery and SD holder were studied. Finally, the circuitry was placed and the motherboard was designed according to the full system placement.

#### 2.3.1. Sensor Placement

Several studies have focused on load sensors and analysed their placement on hands. Some authors have reviewed the number of times a sensor location was used in different settings [29]. Other authors who have studied applications similar to the one in this study give a more specific sensor layout for different sensor typologies used for their devices [30,31]. There are also studies with similar applications but different sensors, such as piezoelectric sensors [32].

A review of the literature shows that the fingertips and mid-finger placements are the most commonly used hand regions. This is related to the way humans take and interact with objects. For pinch grip operations, four and five sensors attached only to distal phalanxes are used.

From this analysis of the literature it was concluded that sensor locations should meet the following requirements:

- Sensors should be placed on regions of the hand that are exposed to load according to the object of the study.
- Sensors must be distributed according to the operations under evaluation.
- Sensors must be placed in strategic regions that do not restrict user movement.

In order to complement the information found in the literature, a user-case experiment was designed and carried out to decide where sensors should be placed. The aim of this experiment was to see which hand regions were used for placing the pieces on assembly lines, and thus, to make a decision about sensor arrangement in the prototype.

The elements involved in the experiment were the following:

- A custom support made of extruded polyethylene in which several holes were drilled to simulate the supports where the pieces should be placed.
- Real pieces from the Volkswagen assembly lines that were painted beforehand with different acrylic paint colours.
- White gloves that the participants wore during the experiment for placing the painted pieces in the supports.

There were six volunteer participants; three males and three females. The average age of the participants was 25 years old. All of them were right-handed.

**Figure 4:**
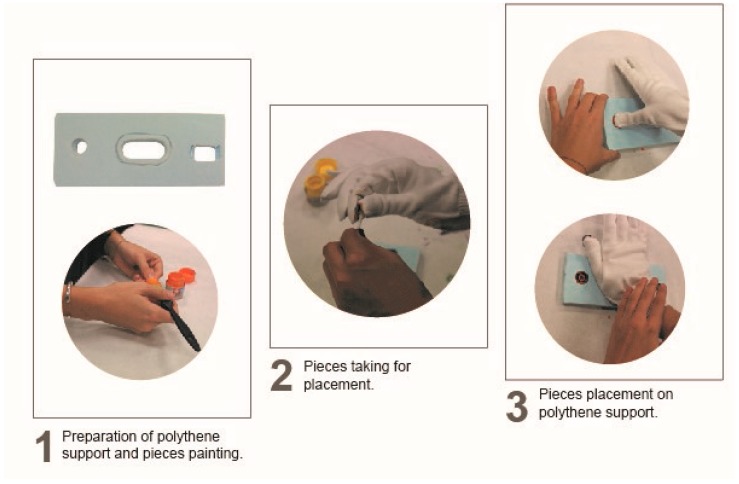
Sensor arrangement experiment.

The experiment was developed following the process shown in Figure 4. First, all the pieces were painted with the acrylic paints (1). A different colour was chosen for each piece in order to identify which hand region was used with each piece. Once all the pieces were painted, volunteers put on the glove and picked up the different pieces (2). Volunteers then placed the pieces in the extruded polythene support (3). Finally, volunteers took off the glove and wrote their name inside it. As a result of this experiment, the hand regions used for placing the different pieces were easily seen thanks to the stains that the paint left on the gloves (see Figure 4).

**Figure 5:**
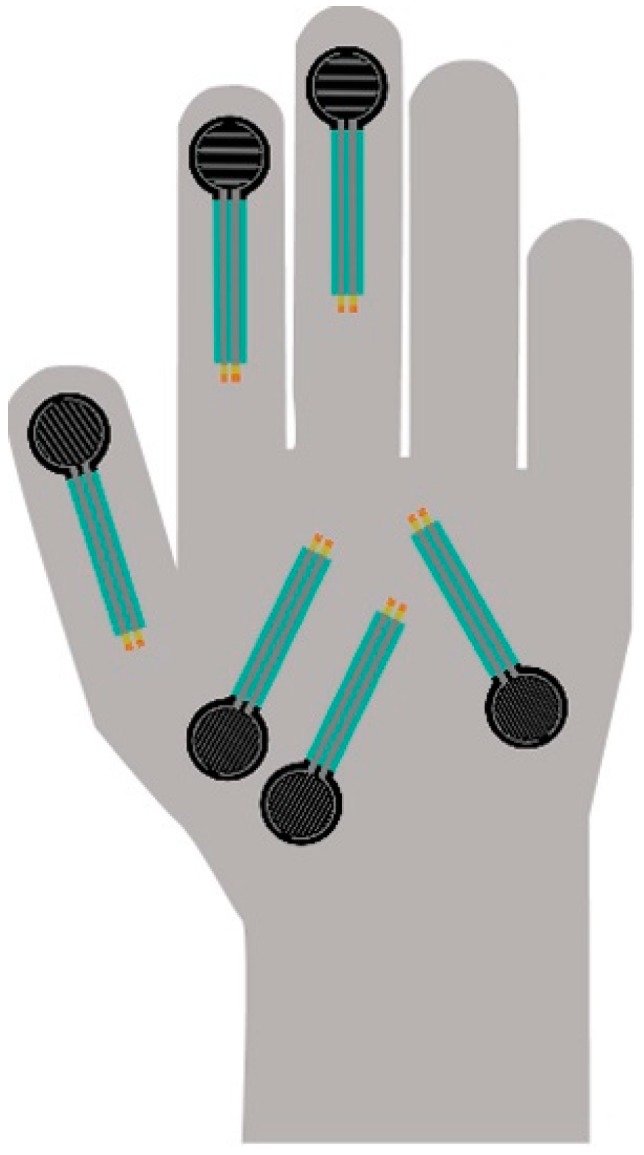
Final sensor arrangement after sensor placement study.

The results of these tests were analysed to decide: (1) how many sensors are necessary, and (2) where they should be placed for monitoring both tasks. The following results were obtained from these user tests. Regarding contact-grip movement, differences between men and woman were found. The palm region is used much more by women than by men, who used the thumb or the index finger instead of the palm. For the distal-compression task, the fingertips, index finger and the thumb were commonly used. In this case, differences between men and women were also found. First, women used the index finger much less than men. This is most likely due to the difference in force between men and women in contact-grip tasks. The middle finger was much less used, although for precision movements it was used in some cases, even though not much force could be exerted with that finger. As far as the authors are concerned, this fact has not been previously identified in other studies. Summing up all the results, and in order to meet the demands of diversity, six main hand regions were identified for sensor placement: three sensors were placed at the thumb (sensor 5), index (sensor 4) and middle fingers (sensor 3), and three sensors at the palm region (sensors 0 + 1 + 2). With this arrangement, the glove can be used interchangeably by all kinds of users and their movements will be properly tracked. The final sensor layout is shown in Figure 5.

#### 2.3.2. SD Breakout Board and Battery Placement

Different hardware configurations of the components were analysed. The components were: a micro SD board, an Arduino microcontroller and a battery holder. For this preliminary study, flexible PCB was not considered due to cost requirements. This initial exercise was important for defining the component size, shape and other facets that would influence the placement of components.

**Figure 6:**
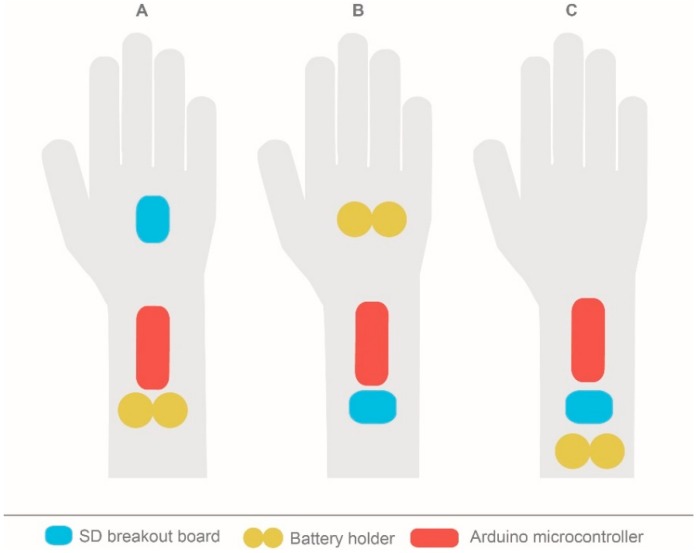
Setup options for the three main components: SD breakout board, battery holder and Arduino microcontroller.

Three different cases were defined, as seen in Figure 6.

- Case A: The SD breakout board is placed on the back of the hand and the microcontroller and battery holder are placed on the forearm.
- Case B: The battery holder is placed on the back of the hand and the microcontroller and SD breakout board are on the forearm.
- Case C: All the components are placed on the forearm.

The three cases influenced the ergonomics as well as the prototype’s performance and fabrication. Placing the components on the wrist or on the back of the hand could be comfortable or uncomfortable for the user. At the same time, different arrangements could make the fabrication of the conductive tracks more difficult, which could also affect the final performance.

For case A, this component placement configuration may affect the circuit design since three elements must be connected and the greater the distance between them, the more difficult it is to design the conductive tracks. Moreover, having the SD breakout board placed on the back of the hand could also affect comfort and the adjustment of the hand.

For case B, similar conclusions can be obtained since the only difference is that the battery holder is on the back of the hand instead of the SD breakout board. The advantage in this case is that the circuit is much easier since it has fewer connections between battery holder and microcontroller than with the SD breakout board.

Finally, in case C, although the configuration increases the forearm final shape, the circuitry design is much easier than in the previous cases. Additionally, having all the components placed on a non-articulated body region is more comfortable.

Hence, the best case was C. Within this configuration, there are multiple options for placing the other components.

#### 2.3.3. Circuitry Placement

The circuit (cable, conductive thread) can be considered another hardware component. In order to design the track configuration, it is important to know the specific placement of the components since the circuitry may change from one case to another. Based on the selected case C, several options were explored.

**Figure 7:**
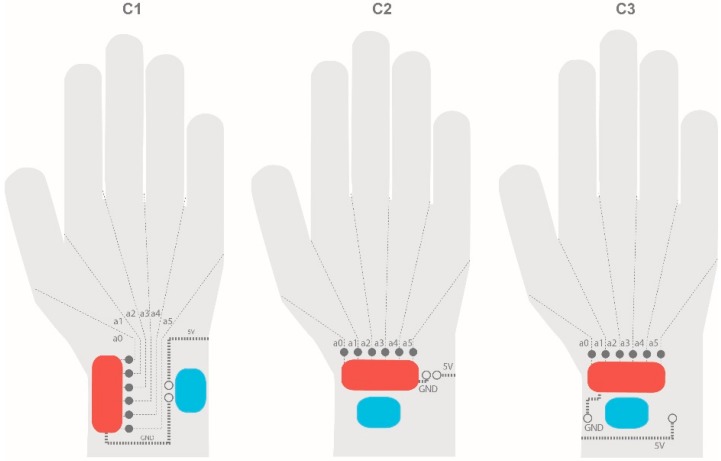
Different component arrangements and circuits for the selected case C based on components orientation.

Figure 7 shows the different cases that were analysed.

Each case (C1, C2, and C3) had a different circuitry configuration, so it was important to construct a low-fidelity prototype and then compare the advantages and disadvantages of each option.

Case C1 places the Arduino microcontroller and battery holder vertically and the input pins are in the middle. Case C2 uses the horizontal configuration and the input pins are in the upper side of the elements. Case C3 has the same configuration as case C2, but with a different input pin placement.

For C1, different circuitry options were drawn. The first case consists of connecting sensor analogue inputs along the back of the hand. Then, they go around the wrist and connect to the board. 5V tracks go along the back of the hand and to the board.

C2 is the opposite of the first case. The analogue inputs go along the palm side of the hand and 5V tracks go along the back of the hand. The analogue inputs are less likely to be short-circuited since the hand cannot be bent upwards, making this configuration a good option for our application.

C3 is very similar to C2, but it traces the conductive trails parallel to the input pins (without going around the wrist).

Both C2 and C3 present more advantages in relation to hardware since the configuration of the motherboard is easier and smaller than in C1. Given the internal circuit, C3 is the easiest option to manufacture, and so it was selected for our project.

#### 2.3.4. Motherboard Design and Placement

Board design was based on the above circuitry design, where C3 was the most suitable circuitry and motherboard connection design for the project. Thus, analogue pins were placed vertically in the board and ground, and source inputs were placed horizontally.

- Board dimensions: The first parameter decided upon was the width and the length of the board, taking into account standard wrist measurements.
- Board fixing: Able to fit and remove easily.
- Board temperature control: Board temperature must be managed while the glove is being worn.

Taking into account standard measures for wrists and forearms [33], a 35 mm wide and 60 mm long board was chosen. In this case, the width would be a constraint since it directly affects users’ comfort. The final configuration was manufactured with a micro-milling machine and FR4, a composite material made of fiberglass and copper.

#### 2.3.5. Power Source

Several factors had to be kept in mind regarding the power source. First, the energy consumption of the entire system and the working time needed for the specific application determined which power source typology was needed. The dimensions of such sources were defined depending on the energy supply needed. In this case, 6V were needed for two hours of working time. As it was necessary to have a rechargeable battery, a lithium-ion power bank was selected. A textile battery holder was selected to position the battery. The entire system was placed on the upper arm using a holder similar to that used by runners to hold a mobile phone in place while running.

#### 2.3.6. Final Arrangement

Once all the components were selected and placed, the final configuration was decided. It consisted of two main layers and another textile protective layer. Different layers were designed to prevent short-circuits with conductive tracks. Thus, it was decided that the tracks from the 5V connecting tab would be made in the inner layer of the palm side of the glove and the tracks from the analogue signal, from the inner layer to the outer layer of the back of the glove.

**Figure 8:**
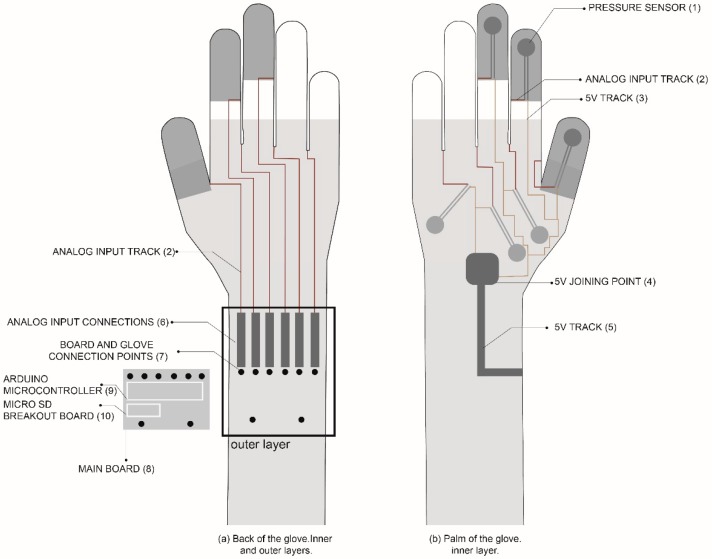
Final glove arrangement.

As shown in Figure 8, the sensors (1) were placed in the inner layer of the palm of the glove. The space between the fingers was used to run the analogue entrance tracks (2) to the layer on the back of the glove. 5V tracks (3) were connected to a common point made of conductive textile (4) that leads directly to the main board through a conductive track made up of a conductive ribbon (5). This conductive ribbon was also used for the analogue input connection (6) in the outer layer on the back of the glove. Thus, the inner and outer layers were connected by this sewn-in ribbon. On the analogue input connections (6), connection points between the board and the glove were sewn in. Those points were made of connection snaps to connect the glove with the main board (8). The Arduino microcontroller (9) and the Micro SD breakout board (10) were placed on the main board. The options of placing the microcontroller and SD card unit with the battery were discarded to provide a more compact solution. Since this glove is meant to be used in real assembly lines, it is important to work on compact solutions to prevent snagging on other hand tools or car parts. Finally, to give more protection to the sensors, a cotton textile layer was sewn over them.

## 3. Results: Delivery Phase

Once the final arrangement was defined, the final prototype design and manufacture were completed. The prototype was then tested on different volunteers. Three of them were internal volunteers from the research institution and another three were workers from Volkswagen Navarra. This experiment was conducted in a laboratory setting.

### 3.1. Final Prototype Design

The fabrication process was based on the same steps defined in the final configuration. Before starting to manufacture the glove, some important requirements were defined, as shown in Table 1.

**Figure 9:**
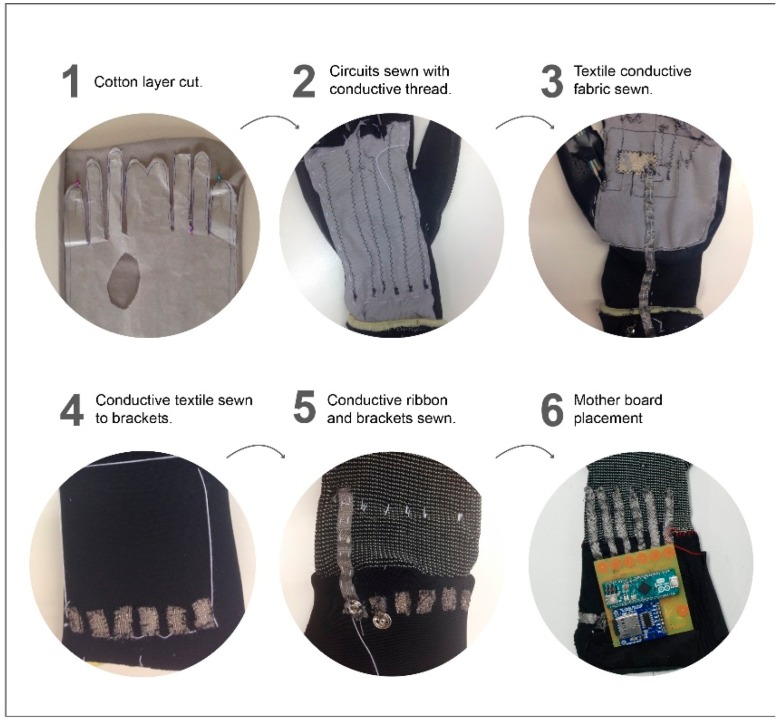
Glove manufacturing process.

**Figure 10:**
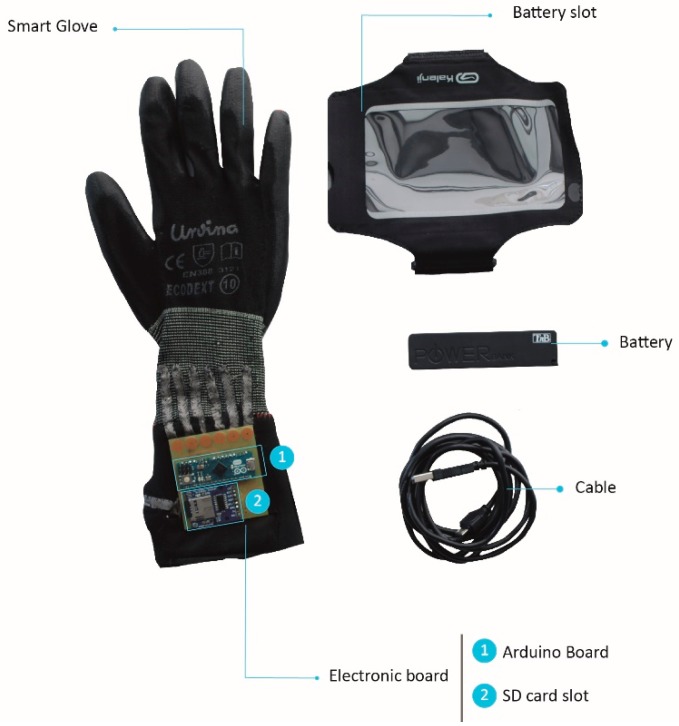
Final glove prototype.

The manufacturing process can be seen in Figure 9. First, (1) the cotton layer must be cut to fit the working glove. The shape should cover both the front and back of the hand with enough fabric between the fingers to keep continuity in the circuits. (2) Circuits were sewn in with normal thread. Before sewing with conductive thread, different configurations were tried on the sewing machine. (3) A textile conductive fabric was sewn into the palm to connect all the 5V entrances and another textile conductive ribbon was sewn in and run to the main board (4). Once the circuits were made, they were provisionally sewn to the glove with normal thread to see where the rest of components should be placed. (5) The glove was turned over and new conductive ribbons were sewn in to connect the analogue entrances with the main board. Then, conductive brackets were sewed onto these conductive ribbon textiles. (6) The main board was made using a milling machine and conductive brackets were soldered to the main board. The final prototype and main components are shown in Figure 10.

### 3.2. Test with Internal and External Volunteers in Lab and at VW Plant

**Figure 11:**
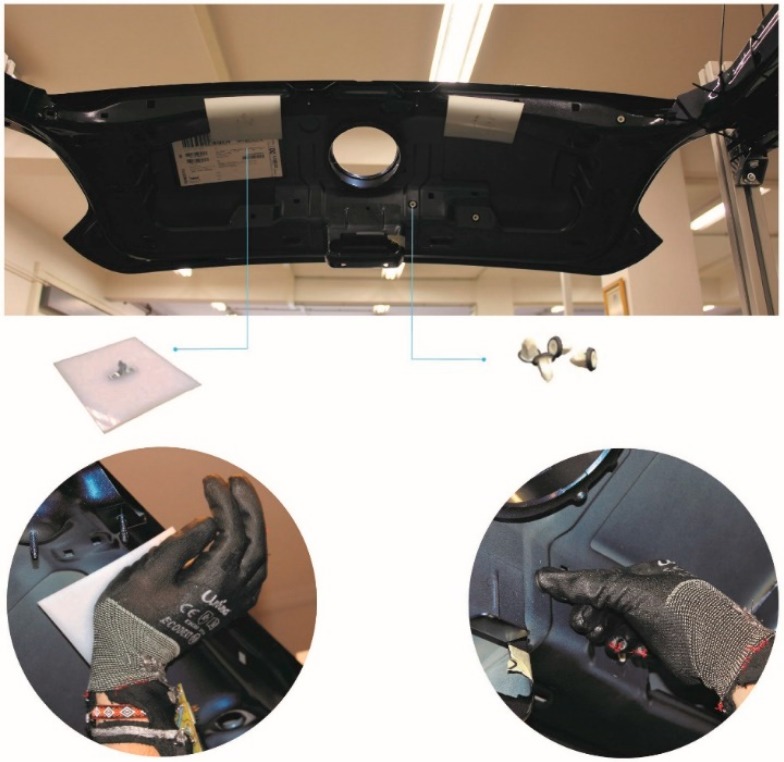
Prototype testing in a real rear door.

In order to assess whether the final design meets the user’s needs, a series of tests was carried out both internally in the laboratory and externally, at the factory. The testing scenario and some of the test tasks are shown in Figure 11.

The test consisted of two parts. In the first, all volunteers were asked to wear the glove and make some static movements to analyse the ergonomic feedback when wearing the glove [31]. After finishing the static movements, volunteers were asked to assemble some plastic pieces on the outside of a rear door of a vehicle. The movements that the volunteers performed for these operations were contact grip and distal compression as shown in Figure 11.

In the laboratory test, the door was attached to a metallic structure in order to mimic the position of the door on the assembly line. There were three subjects involved in this experiment and all of them repeated the experiment 3 times. Thus, in total there were 9 measurements to analyse. After the tests, they were asked to fill out a questionnaire about the glove. The answer for each question follows a Likert scale from 1 (totally disagree) to 7 (totally agree). The same procedure was followed with five external volunteers.

**Figure 12:**
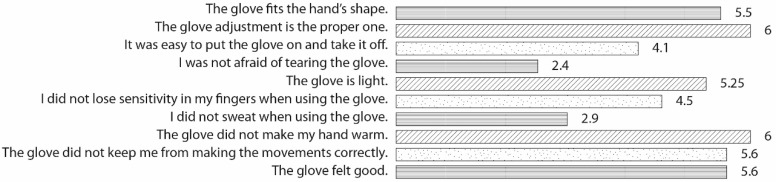
Users mean values for ergonomic questionnaire.

The average results from the both internal and external volunteers regarding the questionnaire are presented in Figure 12. As can be observed, the volunteers felt the glove fit the hand’s shape (5.5/7) and that the glove adjustment was the proper one (6/7). They felt it was light (5.25/7) and they did not feel warm (6/7) but some of them sweated (2.9/7). They agreed that the glove was easy to put on and take off (4.1/7). Sensitivity was positively rated (4.5/7), and they felt that the glove did not affect their ability to carry out the required movements. In general, people thought the glove felt good (5.6/7), although some of them were afraid of tearing the glove (2.4/7).

**Figure 13:**
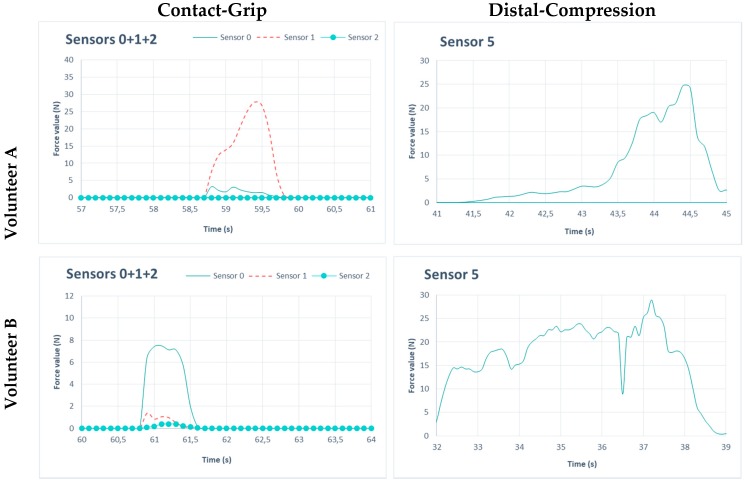
Sensors performance during the assembly tasks.

As far as sensor operation is concerned, the measured data from the sensors were within the range of maximum values per task provided by Volkswagen in Table 2. Figure 13 shows the operation of sensors for the two assembly tasks studied in this article for two volunteers. The contact grip is characterized by punctual contact (duration of 1 second) just as the volunteer was applying the load. The distal-compression is characterized by extended movements so the results show force values for a longer period of time. Higher values were recorded for the contact grip task than for the distal compression task. In terms of gender differences, in both movements force values were greater for men than for women, as shown in Figure 13. However, for injury risk, the maximum values for both movements are far from the maximum values provided by Volkswagen. This means that, according to referent model values, there is no risk of injury.

## 4. Discussion

Wearables are gaining widespread use, and technologies are making it possible to monitor human physical activity and behaviour as part of connected infrastructures. The design process for wearables requires a holistic view of all the factors involved, such as technology, ergonomics, human factors and validation techniques. It is then easier to go deeper into the particular steps that are most relevant to the specific problems that must be solved for different applications. This operating dynamic is easier if a human-centred design process is followed from the very beginning. Moreover, by following this methodology, personalised technology can be obtained, and thus, the integration of flexible technology into the glove is easier since it is based on both user and contextual requirements.

Design requirements should be defined taking into account both the technical components and human aspects. Therefore, the first stage is to create an embodied wearable, benefitting from the nature of various materials and making them flexible and comfortable. In order to meet these requirements several related works can be considered [32,34,35].

This section discusses the importance of a human-centred design during the steps defined in the methodology: the discover phase, the define phase, the develop phase, and the delivery phase.

Several starting specifications were given for our project, such as keeping the original glove used by the workers or that the glove should not include any wireless communication due to IT security reasons. Such specifications usually refer to economic issues or are related to the specific context of use and company preferences and internal standards. Sometimes the user explains the solution they need but not the problem they have. Therefore, it was important to define the brief in collaboration with the company using observational techniques which allowed the initial brief to be checked against the real situation. Once the brief was defined, an ergonomic study was conducted.

In the ergonomic study, sensor arrangement and design requirements were defined. The sensor arrangement was determined by a low-fidelity prototype that helped the design team to make decisions about the hand regions that were used during the tasks. Once this step was finished, the ergonomic requirements were defined. This stage was crucial for the development of the overall project. The prototype was built based on previous studies found in the literature, and it was then complemented by some specific experiments designed by the authors. Whenever a prototype was updated for any ergonomic or human-factor reason, a new ergonomic requirement was added. This procedure allowed us to create a complete table that could be used as a reference model, not only for the glove design but also for designing the ergonomic questionnaires that were administered in the validation stage. Moreover, these specific requirements led the project to search for specific technology options that were completely related to the ergonomic requirements, such as flexibility, comfort, heat protection etc. [36].

The design accommodated a wide range of individual preferences and abilities. The use of the designed wearable should be easily understandable, regardless of the user’s experience, knowledge or communication capabilities. The design communicates information effectively and minimises hazards and adverse consequences. These aspects allowed us to define some ergonomic requirements that formed the basis for the glove design.

In the case of this study, different prototypes were used to check different ergonomic aspects. First, one prototype was built in order to see where the components should be placed; a second one was made to determine the sensor arrangement, and a third one, to examine the performance and test the ergonomics of the glove. In these stages, it was in our interest to include volunteers rather than waiting to bring them in after the building of the high-fidelity prototype was completed.

As a result of the first preliminary test, some changes were made not only to the glove but also to the experimental design. This allowed the team to improve the ergonomics and the procedure of the experiment with internal users.

Finally, and as future lines of investigation, the research team identified areas of improvement for the smart glove design after analysing the results of the questionnaire. First, we considered that it was important to give more instructions when putting on and taking off the glove. Some extra apertures were made in the glove to prevent the accumulation of sweat. In order to relieve the fear of tearing the glove, robustness was improved by adding extra layers inside the glove.

## 5. Conclusions

Regarding the design process, establishing an adapted Double Diamond Modelled to the discovery of several aspects related to human-centred design. First, there are several ways of involving users in the process. These include direct techniques such as meetings or questionnaires, and indirect techniques such as behavioural observation or examining users’ interactions with prototypes. From the meetings that took place during this study, we can conclude that they were useful for identifying common objectives with stakeholders, and even more importantly, for involving them in final decisions. Consequently, communication between the design team and stakeholders was more effective and we worked cooperatively. The questionnaire results showed that they captured information about the ergonomics as well as user satisfaction.

Regarding the behavioural observation, this involves learning about people’s behaviour and activities as they occur in a real-world setting rather than in a controlled environment. In our study, this allowed us to see how the real conditions of everyday interaction impact on how the gloves are used, and in that way, to better define the brief. In short, through the written diaries, we verified that the original problem was correctly identified. Finally, observing how users interact with prototypes, their usability and functionality can be tested. In our case, we checked that participants knew how to use the prototype and that they were able to do their tasks comfortably.

From the results of the internal users’ test, it can be concluded that the objectives of this study were fulfilled. The project targets as defined in the objective section are considered below.

- Integrating the components based on user requirements and application context. Thanks to the process that was followed, user requirements (sensor placement and glove adaptation to different sizing) were considered. Additionally, the company had internal standards that needed to be taken into account such as using a glove currently used on assembly lines to protect workers’ hands. This led the team to develop new processes to integrate flexible technology into an existing glove.
- Designing a prototype suitable not only for laboratory experiments but also for being used in real assembly lines. The preliminary test and observational techniques led the design team to design a glove that was suitable for assembly lines. Based on the results of the first questionnaire, some modifications were made and this led to improved glove performance. At this stage of the research, we focused on functionality and ergonomics without taking into account other aspects of the wearable such as usable life span, which is more related to the industrialization of this device.

Even though the functional results were satisfactory, it was not enough to guarantee good performance and complete user satisfaction. User interaction during the design process is crucial for acceptance of the designed devices and for optimising the time and cost of the design process. Wearable technologies are still complex, and user experience and the use cycle must be improved even further. The role of prototypes in fulfilling these aspects is crucial, and a methodology based on human factors that determines the function of different prototypes during the process can be further developed. This can help designers to improve the results of their work.

## Figures and Tables

**Table 1:** Glove manufacturing requirements.

Component Integration	Isolation	Component Protection
Components should not be easy to remove accidentally.	Cables and components should not short-circuit.	All the electronic components should be protected.
The joints should be strong and flexible.	Sensor entrances should be separated enough so as not to short-circuit.	Hand movements should not affect component life.
Components should be placed taking into account final position of controlling board	The probability of short-circuits must be analysed in all hand positions.	The glove should be tested in a real working environment to ensure correct performance.
Component position should reduce conductive track length		

**Table 2:** Maximum force values during the internal and external test for men and women.

Contact Grip			Distal Compression		
Men	Women	Max. Volkswagen	Men	Women	Max. Volkswagen
38 N	26.5 N	115 N	34.2 N	24.7 N	55 N

## References

[1] Sicchio K., Guler S.D., Gannon M. (2016). A Brief History of Wearables. Crafting Wearables.

[2] Sazonov E., Neuman M.R., Sazonov E. (2014). Wearable Sensors.

[3] Plamondon A., Delisle A., Larue C., Brouillette D., Mcfadden D. (2007). Evaluation of a hybrid system for three-dimensional measurement of trunk posture in motion. Appl. Ergon..

[4] Nordander C., Balogh I., Mathiassen S.E., Ohlsson K., Unge J., Skerfving S. (2004). Precision of measurements of physical workload during standardised manual handling. Part I: Surface electromyography of m. trapezius, m. infraspinatus and the forearm extensors. J. Electromyogr. Kinesiol..

[5] Dipietro L., Sabatini A.M., Dario P. (2008). A survey of glove-based systems and their applications. IEEE Trans. Syst. Man Cybern. Part C Appl. Rev..

[6] Dobkin B.H., Dorsch A. (2011). The promise of mHealth: Daily activity monitoring and outcome assessments by wearable sensors. Neurorehabil. Neural Repair.

[7] Wang C. (2018). Personal PIN Leakage from Wearable Devices. IEEE Trans. Mob. Comput..

[8] Pantelopoulos A., Bourbakis N.G. (2010). A Survey on Wearable Sensor-Based Systems for Health Monitoring and Prognosis. IEEE Trans. Syst. Man Cybern. Part C Appl. Rev..

[9] Olguín D.O., Waber B.N., Kim T., Mohan A., Ara K., Pentland A. (2009). Sensible organizations: Technology and methodology for automatically measuring organizational behavior. IEEE Trans. Syst. Man Cybern. Part B Cybern..

[10] Chan M., Estève D., Fourniols J.-Y., Escriba C., Campo E. (2012). Smart wearable systems: Current status and future challenges. Artif. Intell. Med..

[11] Liang T., Yuan Y.J., Member S. (2016). Wearable medical monitoring systems based on wireless networks: A Review. IEEE Sens..

[12] Leire Frances A.C., Morer P., Rodriguez M. (2018). Revisión de la tecnología wearable y su aplicación en guantes inteligentes. DYNA Ing. Ind..

[13] Schmuntzsch U., Sturm C., Roetting M. (2014). The warning glove—Development and evaluation of a multimodal action-specific warning prototype. Appl. Ergon..

[14] Stiefmeier T., Roggen D., Ogris G., Lukowicz P. (2008). Wearable Activity Tracking in Car Manufacturing. IEEE Pervasive Comput..

[15] ProGlove. http://www.proglove.de/.

[16] XSens, Xsens MVN. https://www.xsens.com/products/xsens-mvn/.

[17] Bae Systems Q-Sight® Helmet. http://www.baesystems.com/en-uk/product/qsight-helmet-mounted-displays.

[18] Vandrico. http://vandrico.com.

[19] Teksan. https://www.tekscan.com/pressure-mapping-sensors.

[20] Tessarolo M., Luca Possanzini L., Campari E.G., Bonfiglioli R., Francesco Saverio Violante F.S. (2018). Adaptable pressure textile sensors based on a conductive polymer. Flex. Print. Electron..

[21] AnthroTronix. http://www.anthrotronix.com/.

[22] Hussain T. (2004). Musculoskeletal symptoms among truck. Occup. Environ. Med..

[23] Zare M. (2015). Evaluation of ergonomic approach and musculoskeletal disorders in two different organizations in a truck assembly plant. Int. J. Ind. Ergon..

[24] Nur N.M., Dawal S.Z., Dahari M. The Prevalence of Work Related Musculoskeletal Disorders Among Workers Performing Industrial Repetitive Tasks in the Automotive Manufacturing Companies. Proceedings of the 2014 International Conference on Industrial Engineering and Operations Management.

[25] UNE 1005 (2003). Safety of Machinery-Human Physical Performance—Part 2: Manual Handling of Machinery and Component Parts of Machinery.

[26] International Organization for Standardization 11226 (2000). Ergonomics—Evaluation of Static Working Postures.

[27] International Organization for Standardization 11228 (2003). Ergonomics—Manual Handling.

[28] Design Council. https://www.designcouncil.org.uk/news-opinion/design-process-what-double-diamond.

[29] Reinvee M., Jansen K. (2014). Utilisation of tactile sensors in ergonomic assessment of hand–handle interface: A review. Agron. Res..

[30] Kong Y., Freivalds A. (2003). Evaluation of meat-hook handle shapes. Int. J. Ind. Ergon..

[31] Reid C.R., Nasa L.M. Evaluate Potential Hand Injury Risk Factors. Proceedings of the International Conference on Environmental Systems.

[32] Edmison J., Jones M., Nakad Z., Martin T. Using piezoelectric materials for wearable electronic textiles. Proceedings of the Sixth International Symposium on Wearable Computers.

[33] UNE-EN International Organization for Standardiyation 7250-1:2010 (2017). Basic Human Body Measurements for Technological Design—Part 1: Body Measurement Definitions and Landmarks.

[34] Tongrod N. A Low-Cost Data-Glove for Human Computer Interaction Based on Ink-Jet Printed Sensors and ZigBee Networks. Proceedings of the 4th IEEE International Symposium on Wearable Computer.

[35] Bordel B., Sanchez de la Rivera D., Sanchez-Pickot A. (2016). Engineering, Building unobtrusive wearable devices: An ergonomic cybernetic glove. J. Internet Serv. Inf. Secur..

[36] Francés-Morcillo L., Morer-Camo P., Rodríguez-Ferradas M.I., Cazón-Martín A. The Role of User-Centred Design in Smart Wearable Systems Design Process. Proceedings of the 15th International Design Conference.

